# Navigating Paths to Wellness: A Strengths-Based Photovoice Study Conducted with One First Nation in Southern Ontario, Canada

**DOI:** 10.3390/ijerph191710979

**Published:** 2022-09-02

**Authors:** Bryan Tanner, Ningwakwe George, Laura Jane Brubacher, Melody E. Morton Ninomiya, Laura Peach, Sharon Bernards, Renee Linklater, Julie George, Samantha Wells

**Affiliations:** 1 Institute for Mental Health Policy Research, Centre for Addiction and Mental Health, 100 Collip Circle Suite 200, London, ON N6G 4X8, Canada; 2 Saugeen District Senior School, Port Elgin, ON N0H 2C4, Canada; 3 Department of Population Medicine, University of Guelph, Guelph, ON N1G 2W1, Canada; 4 Health Sciences, Wilfrid Laurier University, Waterloo, ON N2L 3C5, Canada; 5 School of Public Health Sciences, University of Waterloo, Waterloo, ON N2L 3G1, Canada; 6 Shkaabe Makwa, Centre for Addiction and Mental Health, Toronto, ON M5S 2S1, Canada; 7 Mental Health, Addiction and Violence Support Program, Kettle & Stony Point Health Services, Kettle & Stony Point First Nation, Lambton Shores, ON N0N 1J1, Canada; 8 Campbell Family Mental Health Research Institute, Centre for Addiction and Mental Health, Toronto, ON M5S 2S1, Canada; 9 Dalla Lana School of Public Health, University of Toronto, Toronto, ON M5S 3B2, Canada; 10 Department of Epidemiology and Biostatistics, Western University, London, ON N6A 3K7, Canada; 11 Department of Psychiatry, University of Toronto, Toronto, ON M5S 3B2, Canada; 12 School of Psychology, Deakin University, Melbourne, VIC 3217, Australia

**Keywords:** Photovoice, First Nations, colonialism, family, culture, well-being, substance use

## Abstract

Research on substance use challenges in First Nations communities is often deficit-focused and can reinforce paternalistic stereotypes that lead to further discrimination. In this article, we report on findings of a strengths-based Photovoice project done in collaboration with a First Nations’ community in southern Ontario, Canada to better understand experiences with substance use challenges in the community. We analyzed interview data collected with seventeen individuals who have lived experience or are supporting a loved one with lived experience with a substance use challenge. Participants described sources of strength that characterized their path to wellness, including strong family and social connections, cultural practices, identity, spirituality, day-to-day activities, and helpful supports and services. Furthermore, participants made several suggestions for improving services, including the need for integrated and flexible systems of care and trustful client-provider relationships. At its core, nurturing wellness involved a transformative process involving social and/or cultural connections. The stories shared by participants demonstrate the unique and varied strengths drawn from by individuals dealing with a substance use challenge.

## 1. Introduction

Research involving Indigenous Peoples in Canada has often focused on poor health outcomes and health disparities that exist between Indigenous and Settler populations. This deficit-focused approach has been cited as contributing to and further perpetuating stereotypes of Indigenous Peoples as being inherently unwell, while neglecting systemic and structural causes of health disparities [1,2]. It is now understood that discriminatory acts and policies undertaken by the Canadian government to assimilate Indigenous Peoples, as well as broader currents of anti-Indigenous discrimination that exist throughout Canadian society, have had and continue to have devastating impacts on cultural practices, identities, and health outcomes for many of the first peoples in Canada [1,2,3]. In contrast to deficit-focused approaches, strengths-based research acknowledges that many Indigenous Peoples continue to thrive despite these many adversities and focuses on strengths within individuals and communities that can be built upon to improve community wellness [1,3,4]. In the present article, we characterize sources of strength and identify areas where services within a First Nations community may be strengthened to promote wellness-oriented outcomes in the context of addressing substance use challenges stemming from the enduring effects of colonialism.

### 1.1. Substance Use in the Context of Colonialism

The lives of Indigenous Peoples in Canada have been shaped by attempts at forced assimilation into Settler society [5]. Though many Indigenous Peoples have been able to preserve core aspects of their cultural identities, they continue to face immense social and cultural disruption due to discriminatory practices and policies designed to eradicate Indigenous ways-of-life, such as residential schooling, forced appropriation of traditional lands, bans on use of languages and cultural practices, and racist family separation policies such as the Sixties Scoop and other child welfare policies [5]. Such events continue to impact Indigenous Peoples and communities generations later through a transgenerational form of traumatic stress known as intergenerational or historical trauma. This phenomenon highlights how historical events experienced by one’s ancestors, such as a parent’s or grandparent’s residential school attendance, may continue to have important implications today by shaping social, cultural, and material factors necessary for sustaining wellbeing [6,7].

As a result of generations of attempted assimilation, many communities face elevated rates of preventable health harms compared to the Canadian population at large [5,8]. In particular, many Indigenous communities are disproportionately affected by substance use challenges [5,9,10,11], which contribute to disparities in substance use-related mortality and morbidity between Indigenous and Settler populations [10], and give rise to social costs such as interpersonal violence and family breakdown [11].

Though these are distressing challenges, it is important to note that a large majority of Indigenous Peoples abstain from most licit and illicit substances [12], despite stereotyping to the contrary. Nevertheless, popular discourse regarding the health of Indigenous Peoples can be laden with paternalistic stereotypes that portray them as heavy users of substances requiring external (i.e., Settler-led) intervention [5,13,14]. Preoccupation with intervening on substance use in Indigenous communities has long been a key aspect of Indigenous-Settler relations in Canada. For instance, the 1869 Enfranchisement Act explicitly banned the sale of intoxicants to First Nations Peoples, and provisions controlling the use of alcohol and other intoxicants were a prominent aspect of the 1876 Indian Act [13]. Scholars have argued that these policies have played a key role in justifying assimilatory intrusions into Indigenous communities (e.g., removing children through residential schooling and other child apprehension schemes), leading to the disruption of social, cultural, and community ties [5,13,15]. Today, the abovementioned stereotypes form the basis for discriminatory encounters that can occur within the Settler-dominated institutions that Indigenous Peoples often must contend with [5], including judicial-legal [15], healthcare [16,17], child service [13], and academic systems [14].

While substance use and associated mental health challenges are a pressing concern within a number of Indigenous communities, many Indigenous Peoples have continued to thrive in spite of these challenges [4]. According to the culture-as-wellness hypothesis [18], the promotion and practice of Indigenous cultural practices and/or identity support community wellness by counteracting the cultural and social disruption that underpins contemporary substance use challenges, including the ongoing impacts of historical trauma [18,19,20]. Gone and Calf-Looking [19] offer several plausible mechanisms for the role of culturally oriented interventions in promoting wellness for those who experience substance use challenges, including “spiritual revitalizations that result from Indigenous ceremonial participation, interpersonal reorientations that yield new and renewed relationships in support of sobriety, and psychological transformations that alter personal identity, motivation, and purpose in service to positive lifestyle change” (pp. 295). More broadly, strong social connections, such as those that often exist within families and communities, may positively impact wellness by providing the support necessary for thriving [21].

### 1.2. Study Aim and Objectives

In a recent scoping review of studies on Indigenous community-based mental wellness initiatives, the authors concluded that Indigenous self-determination from the very outset and engagement throughout were lacking in many of the research studies [22]. Thus, to guide the development of community-based and evidence informed mental wellness programming, research is needed that is strengths-focused and that centres around and is driven by the needs, concerns and perspectives of community members.

This article reports on the findings of a strengths-based Photovoice study conducted with Saugeen First Nation as part of the First Nations Mental Wellness Initiative, collaborative community based participatory research project (CBPR) led by representatives from participating First Nations in Southern Ontario, Canada in partnership with researchers from the Centre for Addiction and Mental Health [23].

As part of a larger project that aims to support communities in developing their own community wellness strategies, this study has two main objectives:

(1) To characterize sources of strength drawn from to navigate a path to wellness by members of an Anishinaabe community who have experienced substance use challenges.

(2) To identify areas where services attending to substance use challenges may be strengthened to promote wellness-oriented outcomes.

## 2. Materials and Methods

### 2.1. Community Context

The First Nations Mental Wellness Initiative was conducted in close collaboration with Saugeen First Nation, an Anishinaabe community centered on lands located near and on the Bruce Peninsula in southern, ON, Canada. As of December 2021, Saugeen First Nation (legal name Chippewas of Saugeen) had a registered population of 1934 [24], with just under 800 living on reserve. Saugeen is a vibrant community characterized by strong social connections and growing industry. The community maintains programs and services that employ many of its residents, as well as people from neighbouring communities, including the Saugeen Amphitheatre, where members can earn certification in stone masonry, a Red Seal Endorsed trade. Sauble Park, a popular destination along the eastern shore of Lake Huron, is privately owned and operated by the Saugeen First Nation Chief & Council. Located on South Sauble Beach, the park is maintained by seasonal park staff such as supervisors, gate workers, and maintenance workers and draws much tourism which contributes greatly to the local economy for Saugeen First Nation and the surrounding area.

### 2.2. The First Nations Wellness Initiative

The goal of the First Nations Mental Wellness Initiative [23] is to generate data to inform local mental wellness strategies that are community led and that target issues identified and prioritized by the community. The research relationship was initiated through an engagement process by the project team leads who reached out to staff at Saugeen First Nation to introduce the Initiative. The project team then travelled to the community and met with staff at the Mino Bimadsawin Heath Centre. It was decided that a formal invitation would be sent to Chief and Council to invite the community to participate in the project. The team was invited to present to leadership who then passed a Band Council Resolution to join the project which established the research collaboration. A data sharing agreement was signed by both parties and a Community Advisory Circle consisting of health professionals and other members within the community was formed. The Community Advisory Circle provided guidance throughout the research process to ensure that the research was grounded in community realities and was relevant and meaningful to the community. During the initial stages of the program, local data collection efforts were undertaken using multiple approaches (i.e., community wide survey; interviews; focus groups) to better understand mental health and substance use challenges, systems of services and supports, and opportunities for building on strengths within the community. Findings were synthesized and presented to community members to identify a priority area for wellness strategy development. 150 community members voted to focus on substance use as the priority area for the wellness strategy. Subsequently, a Photovoice study was conducted to better understand and address substance use challenges within the community.

### 2.3. Photovoice

Photovoice is a CBPR methodology intended to empower groups that have been marginalized within research [25,26]. In Photovoice, participants take photographs that reflect lived experiences of their social and/or material realities. Afterwards, participants reflect on the meanings of these photographs individually with a researcher, and/or in a group discussion, resulting in the creation of rich narratives that reflect the participants’ perspectives [26]. In research with First Nations communities, Photovoice can facilitate the centering of First Nations’ knowledges, worldviews, and modes of knowledge-sharing [27,28]. Furthermore, the methodology has been shown to be effective in engaging hidden and systemically marginalized populations such as individuals living with substance use and other mental health challenges [29,30].

### 2.4. Methods to Centre Indigenous Voices in the Research Process

This study was dedicated to community self-determination from its outset, and study design choices were done to ensure the research process respects Indigenous community sovereignty and liberation from Euro-Western centric research practices [31,32]. Most centrally, the inclusion of a known and well-respected Elder from within the community (NG) as research coordinator was a key attribute in ensuring interviews and data interpretation was done in a way that was respectful and responsive to community/participant events, needs, and experiences. Likewise, this helped to ensure the researcher-participant relationship was founded on a sense of trust in the Elder to keep confidentiality, know families and community contexts, connect people with locally available supports, and advocate for recommendations identified in this study.

Furthermore, the use Photovoice was a key consideration in this process. While Photovoice started as a western scientific methodology, it has several attributes that have helped promote an equitable research process that privileges Indigenous voices. The focus on the participant as a knowledge co-creator can help decolonize research encounters by shifting power towards the participant [33], while challenging the notion of the western researcher as sole purveyor of expertise, a process which has been identified as counter-hegemonic [27]. Relatedly, the use of visual and oral storytelling may facilitate inclusivity of non-western ways-of-knowing better than methods rooted solely in western science (e.g., standardized questionnaires).

### 2.5. Recruitment

Eligible participants included community members ages sixteen years or older who had lived experience with a substance use challenge and/or had supported or were supporting a loved one through a substance use challenge. These participants are, respectively, referred to as people with lived experience (PWLE) and people supporting loved ones with lived experience (PSLO). Participants were recruited through three approaches. First, the field coordinator (NG) approached individuals known to be eligible. Second, members of the Community Advisory Circle as well as frontline care workers suggested potential participants whom they felt could make valuable contributions to the project. Finally, some participants came forward after learning that substance use had been chosen as the priority area for the project.

### 2.6. Data Collection

In total, 19 individuals took part in Photovoice, with 17 completing the process (3 men, 14 women). Five participants were PWLE, five were PSLO and seven participants were both PSLO and PWLE. Participants were invited to a group orientation meeting facilitated by the Project Scientist (MMN) with additional support and guidance from the Field Coordinator (NG) where they were provided with information regarding the study and given an overview of the Photovoice process. Written informed consent was obtained at this point. After orientation, participants were given a camera and provided with three questions to keep in mind when taking their photographs. These were: (1) How has substance use (used by yourself or a loved one) impacted your wellbeing? (2) What helps you cope with stress and challenges in your life resulting from substance use? (3) What strengths does your community have that can be built on to help address substance use?

After completing their assignment, participants met with the field coordinator in private one-to-one sessions to discuss their photographs. We provided a high degree of flexibility, particularly when scheduling the one-to-one sessions, to allow for meaningful involvement of participants who were facing complex issues in their lives. In addition to discussing the three substantive questions posed to participants at the outset of the study, the interviewer also probed for recommendations relating to local substance use initiatives that would be most useful to them. Further, participants were encouraged to share any other thoughts or feelings that came to mind during the interviews related to experiences of substance use or community-specific recommendations. The interviews were audio recorded with consent and transcribed for analysis. The interviews ranged from approximately 9.5 to 80 min in length (average interview time: 42 min).

### 2.7. Analysis

Our analytic approach consisted of an iterative thematic content analysis using a constant comparative method within and across transcripts [34], and was guided by a grounded theory approach [35]. Three researchers (NG, LJB, LP), including the interviewer, independently conducted initial open coding of three transcripts [36]. Then, the researchers met to discuss emergent codes and to formulate a codebook for further analysis [36]. This process was used to ensure intercoder reliability through consensus [37]. As the aim of this project was to develop community-specific wellness strategies, coding was conducted with an eye to developing themes that might have practical benefit to the community. Afterwards, one researcher (LJB) re-coded all transcripts, altering, merging, and expanding codes as necessary to reflect the entire dataset. NVivo 12©, Version 12.1.0 software (QSR International, Burlington, VT, USA) was used for organization and application of codes. Triangulation of themes across transcripts, as well as close collaboration among First Nations and Settler researchers throughout the analysis process, contributed to validity of the analysis [38].

Findings from this analysis were previously shared with community stakeholders in the form of a report, which also included a series of recommendations for strengthening local systems of care for substance use. Authors of this article include First Nations (JG, NG, RL) and Settler (BT, LJB, LP, MMN, SB, SW) researchers.

## 3. Results

### 3.1. Substance Use Challenges: Origins and Community Impact

Although the main focus of this article is pathways to wellness, many participants chose to share their lived experience perspectives regarding the impact of substance use on the community and the origins of these challenges. As such, we chose to include the themes that emerged from these discussions to inform and contextualize understandings of sources of strength and services needed to improve community wellness.

Participants spoke of a high prevalence of substance use within the community: *“I was just introduced to it and just everybody was doing it. Yeah, it just seemed like it was the thing to do around here.”* As a participant shared, the high prevalence of substance use was perceived to be burdensome to the community at large: *“This is a community where I don’t know how many young people that we’ve lost in the last three years. We are that community.”* Moreover, substance use was viewed as a shared social illness which had beset the community: *“My people are sick. Like it’s—there’s no difference of addiction to illness, or, right? Sickness—we have a sickness. That’s how I feel.”*

Those who had lived experiences with a substance use challenge (PWLE) were at different points on their journey to wellness. Some reflected on a challenge they faced decades before, while others were still coping with a reoccurring substance use issue. Those caring for loved ones (PSLO) reported that their loved one’s substance use was often an ongoing concern that they were heavily burdened by. Almost all participants were dealing with emotionally burdensome experiences that were related to theirs or their loved one’s substance use, including the early deaths of loved (e.g., overdose) and experiences of intimate partner violence stemming from substance use issues.

Several participants positioned contemporary substance use challenges within the community as structural in nature, identifying these health concerns as an intergenerational impact of colonialism. In particular, contemporary challenges were attributed to discriminatory institutions such as residential schools. One participant attributed the high prevalence of substance use in the community to intergenerational trauma:

It’s what they call intergenerational trauma. I think about the CAS [Children’s Aid Society] and I think about residential school and when those parents came back, those kids came back. The family part, the part of being a parent, they were never taught that.

Likewise, another participant observed a cycle of trauma across multiple generations in her family:

So like coming back to like my generation, we carry that trauma but we don’t know. So the way I look at it and the way that I’ve learned is OK, both of my mom’s parents went to residential school. But I feel like our kids are drowning just as much as we are.

### 3.2. Sources of Strength: Navigating Paths to Wellness

Though participant narratives were punctuated by experiences of emotional and physical pain, they also described several sources of strength that they drew upon in facing a substance use challenge, including the following main domains: family and social connections; culture, cultural identity, and spirituality; community events and day-to-day practices; and services and supports.

#### 3.2.1. Family and Social Connections

Central to participants’ narratives was the foundational role that family and other close social connections played in helping navigate paths to wellness. For PWLE, this involved the steadfast support that they received from close family. As one PWLE dealing with an ongoing substance use challenge noted, *“They’re actually there for me, compared to so many people I thought were my friends—people I could count on. My parents and certain people from my family are actually there for me.”*

For another PWLE who had faced a substance use challenge earlier in their life: *“If I didn’t have that love connection with my grandmother and my one uncle and my aunt, I don’t think I’d make it—that’s what helped me get off the drugs.”*

In addition to the emotional support that they provided, family could provide material support, such as housing or childcare, as well as facilitate transition into treatment. As one PWLE said,

*“I remember being drunk at my partner’s house and my family having to come and get me. And they said, you need to go, ‘we’re here to take you to treatment, you need to go. We’re here supporting you, you said that you would go, and yeah, it’s time’.”*

Similarly, another PWLE stated, “*my sister pulled me aside and said ‘You know, you need to really start to think about something about your drinking’.*”

In some cases, family members served as an inspiration to abstain from substance use. Many PWLE spoke of the strength they gained from younger family members: *“The fact that I wanted to see my grandsons grow up is the only thing that stopped me from continuing on the way I did or the way I could be.*”

The desire to take care of young family members figured prominently in the narratives of PSLO. Often, the support that a PSLO gave involved caring for the children of their loved one. For PSLO, it was these young family members that gave them strength to continue supporting a loved one through a substance use challenge: “*Yeah, there’s a lot going on, but I continue to do what I do because I love my grandkids.*”

Raising these young children was a central aspect of their narratives, and several PSLO made explicit comments regarding a strong desire to protect young family members from cycles of trauma and substance use within their families. As one PSLO noted, “*I’m trying to protect them and give them a childhood and not that trauma.*” Another PSLO stated, “*I grew up in an alcoholic family. I grew up in that but I grew up knowing that I didn’t want my kids to be raised the way I was raised.*”

Nevertheless, for PSLO, the support offered to a family member going through a substance challenge could take its toll. Many PSLO reported feeling overwhelmed, and that caring for a loved one was all-consuming:

I reached out to counsellors, NNADAP [National Native Alcohol and Drug Abuse Program] programs, other family wellbeing programs, anything that promoted our recovery, it was overwhelming for me. I was feeling hopeless, helpless and giving up and that who do I reach out to, who do I need to support me in my journey to help her?

However, these family members persevered. Speaking to the unyielding support that they offered, one PSLO matter-of-factly stated, *“I grew up in a home where that’s just what you do,”* reflecting the strong values that are modeled in the home.

For some PSLO, the strength to continue caring for a loved one was rooted in feelings of empathy arising from their own lived experiences. Referencing their own experiences with a mental health challenge, one PSLO noted, “*It’s just kind of hard because I want to help people and it’s kind of hard seeing because I know how it is and how it feels to go through stuff like that.*”

#### 3.2.2. Culture, Cultural Identity, and Spirituality

Culture, cultural identity, and spirituality were key sources of strength for many participants. Participants described participating in ceremony, visiting Elders and Medicine People, and learning about First Nations culture as ways that they coped with substance use challenges.

For several PWLE, First Nations cultural practices formed a central aspect of their recovery. As one PWLE noted, “*Ceremony saved my life. It’s what lodges—going there to release the things that I needed to release was really helpful.*” Another PWLE, reflecting on their long road to recovery, stated outright, *“Ceremony and cultural practices have brought me to who I am today.*” For this participant, getting involved in regional healing ceremonies allowed for the formation of meaningful and trustworthy social connections, which provided for an opportunity to heal in a welcoming and non-judgmental environment: “*They didn’t look at me with judgement, right. ‘Oh, she looks like a user, or she looks you know, whatever.’ I didn’t get that there at all.*”

For some, First Nations culture lived day-to-day served as a form of therapy. As one PSLO noted:

But I also find that if I do my crafting, I do beadwork. And I make regalia, not only for myself, but my granddaughter. And other people will come and ask me to do things for them, so that’s what I do. That, again, is another form of therapy for me.

The same participant noted the benefit that participating in ceremonies had on helping her get away from the stresses in her life:

It’s the drum. I can hear the drum. And it almost brings me a sense of peace, calm and belonging. And I think that’s what I needed. I needed that. To be away from all the trouble and everything that goes on in this community.

Similarly, cultural activities based in nature provided participants with a peaceful setting in which stress could be released:

Even still today I like to go in the bush and get connected with Mother Earth and be in the forest and just be one with Mother Earth, and that helps me to unload my garbage that I carry throughout the day.

Culture was an important factor defining relationships within families. Mentions were made of family trips to ceremonies, as well as other opportunities to get younger family members involved in First Nations culture. This was proactive at times; speaking of how much time his grandchildren spent in front of electronic devices, one PSLO stated, *“So I thought if I get them outside and take them hunting and whatnot with those bows and give them another interest, something different to do.”*

However, for some, cultural connections were perceived to be under threat. One PWLE perceived a disconnection from the land within the community. They noted, *“I get depressed about my surroundings. The land, environment, especially being Native, we were respecting the land before a lot more than we do now.”* Similarly, one PSLO recalled an instance in which her young grandson they cared for cut his long hair due to teasing from other youth. However, the participant noted that while they were dismayed that this had occurred, they were proud that he recently began growing his hair out again, as the act represented him taking pride in his First Nations heritage. In this vein, reclamation of cultural practices and beliefs was articulated as a key strength; when speaking of the importance of the values they derived from First Nations practices and teachings, one PSLO noted, *“Now as time goes on we kind of reclaim a little bit more of who we are as people.”*

**Figure 1:**
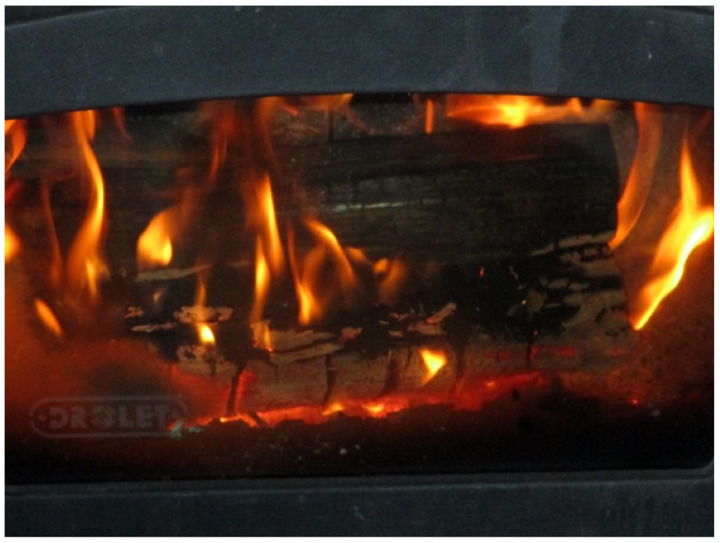
“My Daily Reminder.” This photo is of the participant’s wood stove. It reminds him of learning about the Sacred Fire that helped him put substance use aside.

These teachings became central aspects of the participants’ paths to wellness, imbuing deep spiritual connection into their lives. For the participant who provided the photograph in Figure 1, reconnecting with the Sacred Fire was a central aspect of their path to wellness. Sacred Fires are fires lit to open spiritual ceremonies, for the duration of which the fire is tended to by a Fire Keeper—the Fire is a doorway to the spiritual world, facilitating connections to ancestors that live there, and to whom attendees can send their prayers. For this participant, tending to their wood burning stove, a personal Sacred Fire to which they were Fire Keeper, became a daily reminder of this practice.

Though comments on spirituality most often dealt with First Nations ceremony and cultural practices, a few participants made references to the importance of Christian spiritual orientations. One PSLO spoke of how their strong Christian beliefs help them find strength to continue caring for their loved ones, *“God never wants you to turn your back on anyone that’s hungry. God speaks through many things. That’s where I get my help from is my belief in God.”* Speaking to practices rooted in both Christian and traditional First Nations belief systems, another PSLO stated, *“As long as you’re true to yourself and you don’t have the judgement against anybody and you’re not using your religion or your traditions or your culture, whatever, for hate, I said then you’re winning, right?”*

#### 3.2.3. Community Activities and Day-to-Day Practices

Several participants viewed informal events organized within the community as helpful resources; for example, gatherings that occurred around holidays such as Christmas. One PSLO noted the sense of volunteerism associated with these events: *“I thought that it was very inspiring because it’s an uplifting feeling to know that people that actually go out of their way and volunteer.”* Likewise, one PWLE who was now supporting fellow community members through substance use challenges spoke of the benefits of weekly scheduled events where community members could gather to socialize: *“On Friday nights, we open up the Traditional Room at the Health Centre and provide a pot of soup to community members and welcome anybody that wants to come out and play cards or socialize or whatever.”*

**Figure 2:**
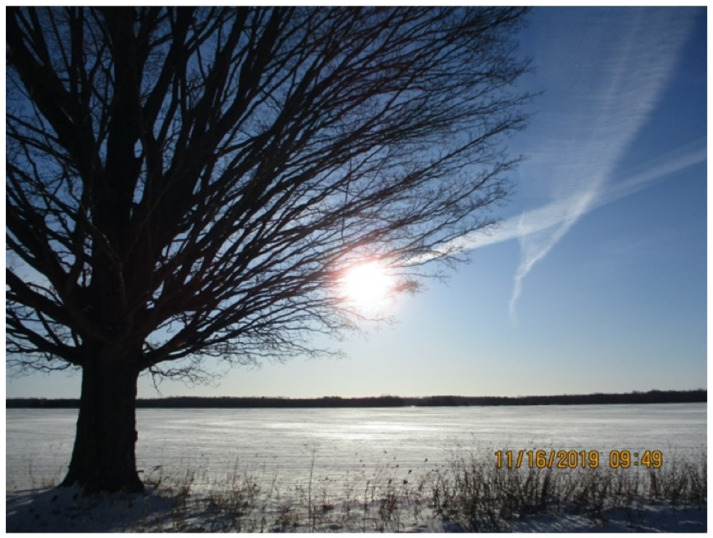
“A New Day.” The participant sees each day as a new beginning. The branches remind her of painful memories that we all carry with us, and the sun is shining through. We need to focus on the beauty that surrounds us.

In addition to these special events, participants often found strength in practices and activities in their day-to-day lives. As shown in Figure 2, being in nature served as a powerful metaphor for remaining positive despite adversity. As described by the PSLO who took the photograph, their painful memories and troubles alongside those experienced by other community members were like branches connected together as one community on the same tree. However, despite the adversities faced by the participant and the community, the sun remained bright, “*bringing in a new day with snow untouched by troubles.*” The benefits of reorienting towards a positive worldview through strong connections to the land and nature were echoed by several participants when describing activities in their daily lives.

For some, physical activity such as exercising at the gym or engaging in sports became coping strategies, helping focus participants in new, positive directions. As one PWLE noted, “*Working out at the gym does help release all of my anger and the issues I have. It helps to meet goals at the gym.*” Similarly, another PWLE spoke of the gym as a place in which they could replace the pain of substance use with a more positive pain associated with physical activity, “*[I]t’s a good sore and it’s a healthy sore.*” Elsewhere, some found benefit in the peaceful solitude provided by activities such as gardening. As one PSLO noted, “*Gardening gives you time to think.*”

Participants noted how employment and education opportunities could help refocus their attention away from the stresses in their lives. As one PSLO working in human services stated:

Sometimes I find that my job takes my mind off of other things. It’s almost like I can escape those problems and those stresses that I have in my own life and I can come to work and I can focus on somebody else’s problems.

For a few PWLE, sobriety signified a hopeful possibility for changes in their lives, such as employment or the option to go back to school.

#### 3.2.4. Services and Supports

Dedicated addictions services available both on and off reserve were seen as helpful in navigating a path to wellness. Formal treatment programming, replacement therapies for opioids, and one-to-one counselling services were examples of services that were drawn upon to help with the physical, psychological, and/or emotional aspects of recovery.

In addition to formal services, informal services such as support groups were drawn upon by PWLE. As one participant noted, *“I attend our Community Addictions Group with other people that have substance problems. It’s just an open group, a check-in group. We get together and we chat, how I feel, talk about other stuff.”*

Likewise, PSLO spoke of the benefits of accessing support groups designed specifically for family members of individuals dealing with a substance use challenge:

There was another program on Thursdays for parents that had addicted children. I went to those kind of groups too and talked about what’s going on in my household and stuff like that and they would help me.

Formal supports were sometimes used in a preventative fashion for children. As one PSLO stated:

It’s been a lot of fighting for our families and trying to make sure our kids have the help they need. So our kids see counsellors. We make sure our kids see counsellors because I know there’s things you can talk to a stranger about [easier] than you can with your own parents, right?

However, gaps in services were also noted, including the need for aftercare. To address these gaps, participants noted that it was only through the actions of individual front line workers who went above and beyond their roles that they received adequate services. Reflecting on this challenge from decades in the past, one PWLE shared:

The only aftercare I got was the fact that the program manager at NNADAP [National Native Alcohol and Drug Abuse Program] wanted to really help us so she took us everywhere. If we had something to do like, you know, maybe spend the whole day, keep us away from the booze. Then we’d go home again in the night.

Though this example was from the past, it was apparent that critical gaps in care were still being covered by professional service providers working beyond their mandate. One PSLO, a service provider within the community, described daily supports she provides to members of her community by giving them much-needed help on her own time for services not covered in program budgets.

### 3.3. Reflections on Community Needs and Services

Participants made several important suggestions regarding the need for new programs and services. Suggestions included a community program where those dealing with addictions could drop in for a meal, prevention services targeting adolescents, proactive outreach for community members known to be facing a substance use challenge, dedicated services for individuals going through difficult initial stages of recovery such as withdrawal, and educational programming on substance use. Furthermore, participants noted that there was a need for a men’s shelter, given that men experiencing homelessness had no available place to stay whereas shelters are available to women.

Speaking to the importance of First Nations culture, one PSLO noted that opportunities for traditional teachings and culturally appropriate services must be made more readily available for community members:

They need to be reminded this is a path you have to walk and we’re not meant to be using drugs and drinking every day and that you can talk to an Elder and they’ll give you wisdom and you can work through your trauma.

In relation to ensuring community supports and services address the needs and concerns of community members, participants highlighted that community members often found themselves in a difficult situation when it came to accessing services that they could trust, noting:

I know counselling is—it’s hard to get around here. It’s hard for people in the community to want to look for a counsellor. People want the help but I think it’s the trust issue. Some people don’t like outsiders coming in and some people can’t talk to people in the community. So it’s—what do they call it? A double-edge sword.

Participants also discussed the role that the community as a whole could play in healing substance use challenges. For example, one participant noted, *“I just wish that everybody could get together and just do a big healing dance—like once a month even. Cause, it is healing for the sick.”* However, lack of knowledge and understanding in the community regarding substance use challenges was cited as a barrier to community-wide change. One PSLO noted that the community at large was unaware of what it is like to care for a loved one dealing with a substance use challenge, whereas her own experiences caring for a loved one had a profound impact on how they view these challenges within the community:

I don’t think that I’d be as strong of an advocate or really pushing for these things if I hadn’t experienced this stuff. I might not understand that, which a lot of people don’t understand, so then when I talk to myself I tell myself they don’t get it, because—it’s not their fault—but it’s because they haven’t had to feel this shit.

A few participants also noted a general lack of work opportunities within the community, which led to filling time in with substance use. One PSLO noted that those dealing with substance use challenges needed opportunities that could provide them with a sense of value: *“They need jobs to make them feel that they’re valued in our community.”*

## 4. Discussion

This article provides rich details regarding pathways to wellness based on the voices of those who have experienced substance use challenges or supported a loved one with such challenges. Using Photovoice methodology, participants shared their perspectives on substance use challenges, sources of strength and pathways to wellness in the face of these challenges, and areas for improving formal services targeting substance use within the community.

### 4.1. Sources of Strength on the Road to Wellness

Consistent with prior research that has shown the importance of strong social ties in engendering wellness for First Nations individuals [21], for many participants, social connections and supports served as essential sources of strength that helped them to cope with substance use challenges and improve their mental wellbeing. The mechanism behind this is likely multifaceted. For example, Cohen [39] suggests two pathways through which social connections may positively influence good health; first, a direct pathway via social integration (i.e., one’s social connections as well as broader social location) that may influence positive health behaviours, and second, an indirect pathway through supportive social interactions (i.e., social support), which can help buffer stressors [39]. As described by participants, the important role of social connections was characterized by instances of direct (e.g., facilitating treatment; family serving as inspiration to stop using substances) and indirect (examples of stress buffering such as the positive impacts of community events; “love connection” with family members) pathways, suggesting both may be fruitful in supporting wellness in the face of substance use challenges.

In addition to social connections, participation in Anishinaabe cultural practices was also cited by some participants as being a major source of strength that contributed to wellbeing. This affirms prior research which has demonstrated the buffering impacts of First Nations culture and/or cultural identities on substance use and mental health outcomes [40,41]. As noted above, reconnecting with First Nations culture and/or cultural identity may serve to alleviate the social and cultural disruption that underpins contemporary substance use challenges [18,19,20]. Moreover, previous research has highlighted that some Indigenous clients seeking help for a substance use challenge may benefit from interventions that integrate Indigenous culture and cultural practices (e.g., land based-activities; ceremonies) [42]. These interventions are thought to be more effective than western oriented approaches to healing by offering holistic care that additionally attends to social, spiritual, and psychological needs of Indigenous clients [42], sentiments that were echoed by several Photovoice participants.

The importance of both social and cultural ties in promoting wellness cannot be understood without acknowledging that the disruption of these ties was a central aspect of the attempted assimilation of First Nations by the Settler government. Discriminatory policies such as residential schooling and other child apprehension schemes were designed to destroy First Nations culture by removing children from their families, thereby disrupting social, familial, and cultural ties across generations, which consequently has given rise to the intergenerational trauma that underlines contemporary substance use challenges [5,13,43]. When viewed within this historical context, individual references to intergenerational trauma, personal and familial histories of substance use, cultural loss and reclamation, and the importance of strong social connections can be understood as personal anecdotes in a broader community history shaped by both exposure to and resistance against a discriminatory program of assimilation.

In this regard, the narratives exemplify what Anishinaabe writer and theorist Gerald Vizenor has dubbed survivance. According to Vizenor [44], stories of survivance entail “an active resistance and repudiation of [Settler] dominance [and] obtrusive themes of tragedy, nihilism, and victimry,” (pp. 11), and instead promote narratives that demonstrate an “active presence, more than the instincts of survival, function, or subsistence” (ibid). Contrary to assumptions of Indigenous victimry, the stories and perspectives shared by participants detail a community at work adapting to address a pressing challenge, countering assumptions of helplessness and inevitability in the face of substance use challenges by offering opportunities for hope and wellness founded in strong social, familial, and cultural bonds formed locally.

According to Kirmayer [45], “[h]ealing involves a basic logic of transformation from sickness to wellness that is enacted through culturally salient metaphorical actions” (p. 34). For First Nations Peoples and communities, this involves a transformative process wherein First Nations culture is drawn from to attend to the social, economic, and cultural upheaval caused by centuries of discrimination stemming from colonization [18,46,47,48]. Healing the impacts of this disruption transcends biomedical binaries of disease and cure, instead focusing on a journey to wellness facilitated by reclamation of cultural identities and connections [46]. According to the First Nations Mental Wellness Continuum Framework [20], a central aspect of this process is ensuring individuals have, “**purpose** in their daily lives; **hope** for their future; a sense of **belonging** within their families, to community, and to culture; and a sense of **meaning** [as] part of creation and a rich [cultural] history.” (pp. ii).

In the present study, a process of healing was broadly enacted consistent with the First Nations Mental Wellness Continuum Framework in two distinct ways. First, social and/or cultural ties were drawn upon to reorient individuals towards wellness. This involved diverse scenarios wherein these connections were used to help buffer health outcomes, be it a substance use challenge specifically, or stress arising from the substance use challenge of a loved one. For those dealing directly with the former, these connections provided critical resources to sustaining wellness, either by promoting or maintaining sobriety. For the latter, these connections provided opportunity to get away from contemporary substance use concerns through purposeful and positive moments of respite. Second, some spoke of these connections in clear preventative terms, seeing social and cultural connections as a way to ensure healthy development for younger family members. These strengths were viewed as opportunities to transform health by instilling new purposeful social roles that are rooted in hope, and characterized by strong connections to culture, family, and community. Thus, the perspectives shared by participants broadly reiterate the key components of purpose, hope, belonging, and meaning enumerated in the First Nations Mental Wellness Continuum Framework as central aspects to the transformative process of healing from substance use.

### 4.2. Suggestions for Strengthening Formal Services

As described by participants, formal treatment services were often an important resource in navigating a path to wellness. However, several areas for improvement were noted. This included the need for wholistic services that consider not only the immediate process of recovery, but also the provision of aftercare, as well as services targeting caregivers and adolescents. It is noteworthy that in Saugeen First Nation, informed by the present findings, a transition house was recently established for people needing accommodation and support within the community both prior to and after receiving substance use treatment.

In line with previous research [49,50,51,52], participants spoke of how distrust could be a significant barrier in seeking help from formal services. For some community members, lack of trust stemmed from service providers being located outside the community. Though not discussed explicitly by participants, a key component of ensuring trust in the provision of health care services with Indigenous clients is a commitment to *cultural safety*. At its core, the provision of culturally safe care requires awareness on the part of (often non-Indigenous) service providers of their own biases and privileges in healthcare encounters involving Indigenous clients [53]. This requires care providers to challenge the inherent power differential that exists between providers and clients, by shifting power over healthcare decisions to the client [53]. Conversely, lack of trust of local care providers was also noted as an issue by participants. Service provision in small and rural communities may be complicated by personal bonds that exist outside of the provider-client relationship; for example, in addition to their clinical relationship, clients and service providers, or staff supporting service providers, may share additional bonds as friends, neighbours, and family, which can lead to concerns regarding the perceived privacy of clinical encounters [52,54]. In these cases, the onus is on service providers to ensure client privacy and confidentiality are protected during the delivery of services. Taken together, this suggests that approaches to reducing barriers must be attuned to the specific care needs of each individual. Flexible and wholistic treatment options should be made available to ensure that the person seeking care can do so in an environment that fosters the development of trustful relationships with service providers.

### 4.3. Study Strengths and Limitations

Photovoice was a key strength of this study, as it allowed participants to take a leading role in the research process rather than serve as passive research subjects. Participants reported feeling proud that the data would belong to the community and be directly used for the community’s benefit, both in the implementation of wellness strategies, and to provide data to inform future proposals concerning community health initiatives. Additionally, participants were grateful for the opportunity to share their stories so that they may inspire others facing substance use challenges. This observation is in line with Wang and Burres’ [26] conceptualization of Photovoice as a participatory form of community needs assessment that is well suited to engaging individuals to take an active role in addressing challenges within their communities.

Previous research has found that Photovoice excels at engaging hard-to-reach and often stigmatized populations who may be wary of sharing their experiences in research settings [29,30]. In our study, the high rate of completion of the Photovoice process (~89%) speaks to the effectiveness of Photovoice in engaging persons experiencing or caring for those experiencing substance use challenges. Nevertheless, as we found in this study, flexibility and compassion is required by researchers and communities interested in implementing similar projects due to the sensitive nature of the subject matter and often-difficult day-to-day experiences of participants. In our study, this was achieved by the involvement of a trusted and well-respected local Elder (NG) as research coordinator, who was well situated within the community and attuned to issues and relationships among the participants and the community at large.

Several methodological limitations are present. We were unable to conduct gender-specific analyses of the Photovoice interview data because the majority of Photovoice participants were women. Additionally, due to the small number of men who took part, important experiences for men in the community related to substance use challenges may be missing from the findings. Limitations related to the impacts of the COVID-19 pandemic are also present. For example, we were unable to hold group discussion sessions where participants could talk about their experiences together due to a sudden state of lockdown in the Spring of 2020. Though participants were able to discuss their experiences in one-to-one interviews, the process of group discussion may have led to the development of a novel set of viewpoints over and above those captured through one-to-one interviews. Finally, the Photovoice project dealt with substance use broadly rather than the use of specific types of substances. As such, we are unable to report on experiences with specific types of substances (e.g., opioids, alcohol, cannabis, stimulants, etc.), which may have provided helpful insights.

## 5. Conclusions

The Photovoice project provided an opportunity for community members to share their lived experiences with substance use challenges, with a strengths-based lens. In doing so, participants highlighted sources of strength that might be supportive for other individuals experiencing these challenges. The insights provided within this article offer several starting points for interventions targeting substance use challenges within First Nations communities that are rooted in First Nations’ strengths. However, our focus on strengths should not hide the burden of substance use felt within many communities. Rather, the aim of this article was to amplify voices of individuals who know such challenges, offering possible paths forward for other individuals facing these burdens. While these voices offer a starting point for addressing substance use within First Nation communities, action is needed from policy makers to redress the structural causes of substance use disparities, including socioeconomic inequities and unequal access to healthcare that sustain substance use challenges.

## Data Availability

All relevant data are contained within the article.

## Abbreviations

CAMH	Centre for Addiction and Mental Health
CBPR	Community based participatory research
PWLE	People with lived experience
PSLO	People supporting loved ones with lived experience

## References

[1] Bryant J., Bolt R., Botfield J.R., Martin K., Doyle M., Murphy D., Graham S., Newman C.E., Bell S., Treloar C. (2021). Beyond deficit: ‘strengths-based approaches’ in Indigenous health research. Sociol. Health Illn..

[2] Hyett S.L., Gabel C., Marjerrison S., Schwartz L. (2019). Deficit-Based Indigenous Health Research and the Stereotyping of Indigenous Peoples. Can. J. Bioeth..

[3] Hyett S., Marjerrison S., Gabel C. (2018). Improving health research among Indigenous Peoples in Canada. Can. Med Assoc. J..

[4] Kirmayer L.J., Sehdev M., Whitley R., Dandeneau S.F., Isaac C. (2009). Community resilience: Models, metaphors and measures. Int. J. Indig. Health.

[5] Allan B., Smylie J.K. (2015). First Peoples, Second Class Treatment: The Role of Racism in the Health and Well-Being of Indigenous Peoples in Canada.

[6] Hartmann W.E., Wendt D.C., Burrage R.L., Pomerville A., Gone J.P. (2019). American Indian historical trauma: Anticolonial prescriptions for healing, resilience, and survivance. Am. Psychol..

[7] Gone J.P., Hartmann W.E., Pomerville A., Wendt D.C., Klem S.H., Burrage R.L. (2019). The impact of historical trauma on health outcomes for indigenous populations in the USA and Canada: A systematic review. Am. Psychol..

[8] Reading C., Wien F. (2009). Heath Inequalities and Social Determinants of Aboriginal Peoples’ Health.

[9] Belzak L., Halverson J. (2018). The opioid crisis in Canada: A national perspective. Health Promot. Chronic Dis. Prev. Can..

[10] Firestone M., Tyndall M., Fischer B. (2015). Substance Use and Related Harms among Aboriginal People in Canada: A Comprehensive Review. J. Health Care Poor Underserved.

[11] Reading J.R., Halseth R. (2013). Pathways to Improving Wellbeing for Indigenous Peoples: How Living Conditions Decide Health.

[12] First Nations Information Governance Centre (FNIGC) (2018). National Report of the First Nations Regional Health Survey, Phase 3.

[13] de Leeuw S., Greenwood M., Cameron E. (2009). Deviant Constructions: How Governments Preserve Colonial Narratives of Addictions and Poor Mental Health to Intervene into the Lives of Indigenous Children and Families in Canada. Int. J. Ment. Health Addict..

[14] O’Neil J.D., Reading J.R., Leader A. (1998). Changing the relations of surveillance: The development of a discourse of resistance in Aboriginal epidemiology. Hum. Organ..

[15] Marshall S.G. (2015). University of Manitoba Canadian Drug Policy and the Reproduction of Indigenous Inequities. Int. Indig. Policy J..

[16] Browne A.J. (2009). Discourses influencing nurses’ perceptions of First Nations patients. Can. J. Nurs. Res..

[17] Goodman A., Fleming K., Markwick N., Morrison T., Lagimodiere L., Kerr T. (2017). “They treated me like crap and I know it was because I was Native”: The healthcare experiences of Aboriginal peoples living in Vancouver’s inner city. Soc. Sci. Med..

[18] Brady M. (1995). Culture in treatment, culture as treatment. A critical appraisal of developments in addictions programs for indigenous North Americans and Australians. Soc. Sci. Med..

[19] Gone J.P., Calf Looking P.E. (2011). American Indian culture as substance use treatment: Pursuing evidence for a local intervention. J. Psychoact. Drugs.

[20] Thunderbird Partnership Foundation (2015). First Nations Mental Wellness Continuum Framework.

[21] Richmond C.A., Ross N.A., Egeland G.M. (2007). Social Support and Thriving Health: A New Approach to Understanding the Health of Indigenous Canadians. Am. J. Public Health.

[22] Venugopal J., Ninomiya M.E.M., Green N.T., Peach L., Linklater R., George P., Wells S. (2021). A scoping review of evaluated Indigenous community-based mental wellness initiatives. Rural Remote Health.

[23] Morton Ninomiya M., George N., George J., Linklater R., Bull J., Plain S., Peach L., Stergiopoulos V., Kurdyak P., McKinley G. (2020). A community-driven and evidence-based approach to developing mental wellness strategies in First Nations: A program protocol. Res. Involv. Engagem..

[24] Government of Canada (2021). First Nations Profiles. https://fnp-ppn.aadnc-aandc.gc.ca/fnp/Main/Search/FNRegPopulation.aspx?BAND_NUMBER=123&lang=eng.

[25] Fosterfishman P.G., Nowell B., Deacon Z., Nievar A., McCann P. (2005). Using Methods That Matter: The Impact of Reflection, Dialogue, and Voice. Am. J. Community Psychol..

[26] Wang C., Burris M.A. (1997). Photovoice: Concept, Methodology, and Use for Participatory Needs Assessment. Health Educ. Behav..

[27] Brooks C.M., Poudrier J. (2014). Anti-Oppressive Visual Methodologies: Critical Appraisal of Cross-Cultural Research Design. Qual. Sociol. Rev..

[28] Castleden H., Garvin T., Nation H.-A.F. (2008). Modifying Photovoice for community-based participatory Indigenous research. Soc. Sci. Med..

[29] Han C.S., Oliffe J.L. (2016). Photovoice in mental illness research: A review and recommendations. Health.

[30] Switzer S., Guta A., de Prinse K., Carusone S.C., Strike C. (2015). Visualizing harm reduction: Methodological and ethical considerations. Soc. Sci. Med..

[31] Smith L.T. (2012). Decolonizing Methodologies: Research and Indigenous Peoples.

[32] Kovach M. (2009). Indigenous Methodologies: Characteristics, Conversations and Contexts.

[33] Jull J., Morton-Ninomiya M., Compton I., Picard A. (2018). Fostering the conduct of ethical and equitable research practices: The imperative for integrated knowledge translation in research conducted by and with indigenous community members. Res. Involv. Engag..

[34] Braun V., Clarke V. (2006). Using thematic analysis in psychology. Qual. Res. Psychol..

[35] Charmaz K. (2006). Constructing Grounded Theory: A Practical Guide Through Qualitative Analysis.

[36] Decuir-Gunby J.T., Marshall P.L., McCulloch A.W. (2010). Developing and Using a Codebook for the Analysis of Interview Data: An Example from a Professional Development Research Project. Field Methods.

[37] O’Connor C., Joffe H. (2020). Intercoder Reliability in Qualitative Research: Debates and Practical Guidelines. Int. J. Qual. Methods.

[38] Creswell J.W., Miller D.L. (2000). Determining Validity in Qualitative Inquiry. Theory Pract..

[39] Cohen S. (2004). Social relationships and health. Am. Psychol..

[40] Gray A., Cote W. (2019). Cultural connectedness protects mental health against the effect of historical trauma among Anishinabe young adults. Public Health.

[41] Spence N.D., Wells S., Graham K., George J. (2016). Racial Discrimination, Cultural Resilience, and Stress. Can. J. Psychiatry.

[42] Rowan M., Poole N., Shea B., Gone J.P., Mykota D., Farag M., Hopkins C., Hall L., Mushquash C., Dell C. (2014). Cultural interventions to treat addictions in Indigenous populations: Findings from a scoping study. Subst. Abus. Treat. Prev. Policy.

[43] Bombay A., Matheson K., Anisman H. (2013). The intergenerational effects of Indian Residential Schools: Implications for the concept of historical trauma. Transcult. Psychiatry.

[44] Vizenor G. (2008). Survivance: Narratives of Native presence. Lincoln, Nebraska.

[45] Kirmayer L.J. (2004). The cultural diversity of healing: Meaning, metaphor and mechanism. Br. Med Bull..

[46] Waldram J.B. (2013). Transformative and Restorative Processes: Revisiting the Question of Efficacy of Indigenous Healing. Med Anthr..

[47] Gone J.P. (2009). A community-based treatment for Native American historical trauma: Prospects for evidence-based practice. J. Consult. Clin. Psychol..

[48] Gone J.P. (2013). Redressing First Nations historical trauma: Theorizing mechanisms for indigenous culture as mental health treatment. Transcult. Psychiatry.

[49] Browne A.J., Fiske J. (2001). First Nations Women’s encounters with mainstream health services. West. J. Nurs. Res..

[50] Burnett K., Sanders C., Halperin D., Halperin S. (2020). Indigenous Peoples, settler colonialism, and access to health care in rural and northern Ontario. Health Place.

[51] Richmond C.A.M. (2007). Narratives of Social Support and Health in Aboriginal Communities. Can. J. Public Health.

[52] Rizkalla K., Maar M., Pilon R., McGregor L., Reade M. (2020). Improving the response of primary care providers to rural First Nation women who experience intimate partner violence: A qualitative study. BMC Women’s Health.

[53] Brascoupé S., Waters C. (2009). Cultural safety: Exploring the applicability of the concept of cultural safety to Aboriginal health and community wellness. J. Aborig. Health.

[54] Werth J.L., Hastings S.L., Riding-Melon R. (2010). Ethical challenges of practicing in rural areas. J. Clin. Psychol. Sess..

